# The PSMD14 inhibitor Thiolutin as a novel therapeutic approach for esophageal squamous cell carcinoma through facilitating SNAIL degradation

**DOI:** 10.7150/thno.46109

**Published:** 2021-04-03

**Authors:** Chao Jing, Xingchen Li, Mengqian Zhou, Shengchi Zhang, Qingchuan Lai, Dandan Liu, Beibei Ye, Linqi Li, Yue Wu, Hong Li, Kai Yue, Peng Chen, Xiaofeng Yao, Yansheng Wu, Yuansheng Duan, Xudong Wang

**Affiliations:** 1 Department of Maxillofacial and Otorhinolaryngological Oncology, Tianjin Medical University Cancer Institute and Hospital, Key Laboratory of Cancer Prevention and Therapy, Tianjin Cancer Institute, National Clinical Research Center of Cancer, Tianjin 300060, China.; 2 Department of Ear, Nose and Throat, Tianjin 1st Centre Hospital, Tianjin 300192, China.

**Keywords:** Esophageal squamous cell carcinoma, Thiolutin, PSMD14, SNAIL, EMT, Chemosensitivity.

## Abstract

Metastasis and chemoresistance are major causes of poor prognosis in patients with esophageal squamous cell carcinoma (ESCC), manipulated by multiple factors including deubiquitinating enzyme (DUB). DUB PSMD14 is reported to be a promising therapeutic target in various cancers. Here, we explored the antitumor activity of Thiolutin (THL), the PSMD14 inhibitor, as a new therapy strategy in ESCC.

**Methods:** Through 4-NQO-induced murine ESCC model, we investigated the expression of PSMD14 in esophageal tumorigenesis. Ubiquitin-AMC assay was performed to evaluate DUB activity of PSMD14 with THL treatment. The effect of THL on epithelial-to-mesenchymal transition (EMT), invasion, stemness and chemosensitivity was detected by using *in vitro* and *in vivo* experiments. Immunoprecipitation and* in vivo* ubiquitination assay were conducted to examine whether THL could impair the deubiquitination and stability of SNAIL regulated by PSMD14.

**Results:** Compared with normal esophageal epithelium, PSMD14 was upregulated in 4-NQO-induced murine esophageal epithelium dysplasia and ESCC tissues. THL could significantly weaken DUB activity of PSMD14. Furthermore, the results of *in vitro* and *in vivo* assays showed that THL efficiently suppressed motility and stemness and increased sensitivity to cisplatin in ESCC. Mechanically, THL impaired the interaction between PSMD14 and SNAIL, then promoted the ubiquitination and degradation of SNAIL to inhibit EMT which plays a crucial role in ESCC metastasis, stemness and chemosensitivity. TCGA database analysis revealed that high concomitant PSMD14/SNAIL expression predicted shorter overall survival in esophageal cancer.

**Conclusion:** Our findings demonstrate for the first time that suppression of PSMD14/SNAIL axis by THL could be a novel and promising therapeutic approach for ESCC clinical therapy.

## Introduction

Esophageal cancer (EC) is one of the most aggressive cancer types with an increasing incidence worldwide 1. As the predominant histological cancer type, esophageal squamous cell carcinoma (ESCC) accounts for ~80% of EC diagnosed 2. Recently, developed systemic therapies have improved outcomes, but the 5-year overall survival rate of patients with ESCC remains at 15%-25%, which is mainly caused by both metastasis and resistance against chemotherapeutic drugs such as cisplatin 1, 3. Therefore, there is an urgent need for exploring effective therapeutic approaches to treat ESCC.

Deubiquitinating enzyme (DUB) 26S proteasome non-ATPase regulatory subunit 14 (PSMD14), also known as RPN11 and POH1, is one of essential components of 19S proteasomal subunit 4. JAB1/MPN/Mov34 (JAMM) domain is crucial for the DUB activity of PSMD14, which cleaves off conjugates of ubiquitin depending on Zn^2+^ 5. Through regulating protein deubiquitination and stabilization, PSMD14 is implicated in various biological processes, including differentiation 6, cell viability 7, pluripotency 8, autophagy 9, DNA break responses 10, 11 and tumor progression 12. In hepatocellular carcinoma, PSMD14 enhanced the activation of TGF-β signaling and tumor metastasis by deubiquitinating TGF-β receptors and caveolin-1 13. Moreover, PSMD14-mediated E2F1 stabilization could promote tumor formation in liver cancer 14. Therefore, PSMD14 is considered to become a promising target for cancer therapy. Inhibitors of PSMD14, however, have not been preliminarily investigated so far.

The epithelial-mesenchymal transition (EMT) is a reversible and highly conserved biological process whereby epithelial cells are transformed into mesenchymal cells 15. Accumulating evidence has suggested that EMT is implicated in tumor progression and metastasis and predicts poor clinical prognosis 16. Tumor cells that have undergone EMT trigger the invasion and metastasis and acquire other new biological characteristics, such as higher resistance to chemotherapy 17-19. Importantly, specific EMT transcription factors (EMT-TFs) contribute to initiate EMT process 15, 20. SNAIL, an EMT-TF with Zinc finger domain, is an essential initiator to trigger EMT by repressing E-Cadherin expression 21. Emerging researches indicate that elevated SNAIL correlates positively with tumor grade, metastasis and poor outcomes 17, 22-25. Furthermore, SNAIL confers resistance to drugs and survival under stress in multiple cancers 17. SNAIL is, thus, a potent therapeutic target. Unfortunately, no drugs targeting SNAIL have been approved for cancer clinical practice until now.

Thiolutin (THL), a zinc chelator, is a disulfide-containing antibiotic and anti-angiogenic compound 26. Minamiguchi *et al.* found that THL inhibited the adhesion of human umbilical vein endothelial cells (HUVECs) to vitronectin by reducing paxillin in HUVECs and suppressed tumor cell-induced angiogenesis* in vivo* 27. Besides, THL could inhibit endothelial cell adhesion by induction of Hsp27 phosphorylation and block both wound-driven and tumor-driven vascular outgrowths by utilizing an *ex vivo* model 28. Recently, Thiolutin was confirmed to inhibit JAMM-domain containing proteases such as PSMD14 by complexing the catalytic Zn^2+^ ion in the active center of the enzymes 29. In this study, we evaluated the efficacy of THL in ESCC treatment via inhibiting deubiquitinating enzyme PSMD14. The compound was shown to reverse the EMT process and increase cisplatin sensitivity *in vitro* and *in vivo*. We further identified that THL exerted its antitumor effects by impairing PSMD14-mediated deubiquitination and stabilization of SNAIL. These findings indicated that it is possible for THL to be a candidate for ESCC therapy.

## Materials and methods

### Cell culture

The human ESCC cell lines KYSE 30, KYSE 140, KYSE 150, KYSE 450 and KYSE 510 were generously provided by Dr. Shimada Y. (Kyoto University, Kyoto, Japan). The EC 109 and TE 1 cell lines were obtained from Shanghai Institutes for Biological Sciences. All the cell lines were maintained in RPMI1640 supplemented with 10% FBS and penicillin (100 U/mL)/streptomycin (100 mg/mL). All cells were cultured under humanized conditions (37 °C, 5% CO_2_) and checked for Mycoplasma contamination regularly. The cell lines used in the article were authenticated by short tandem repeat (STR) genotyping.

### Antibodies and reagents

The antibodies used in this study were as follows: anti-PSMD14 (12059-1-AP) and anti-SNAIL (13099-1-AP) were purchased from Proteintech (Rosemont, IL, USA); anti-PSMD14 (HPA002114) was purchased from Sigma-Aldrich (St. Louis, MO, USA); anti-PSMD14 (sc-100464) and anti-GAPDH (sc-365062) were obtained from Santa Cruz (Dallas, Texas, USA); anti-SNAIL (#3879), anti-E-Cadherin (#3195), anti-N-Cadherin (#13116), anti-Vimentin (#5741), anti-Cortactin (#3503), anti-PARP (#9542), anti-Caspase-3 (#9662), anti-Caspase-9 (#9502), anti-Ubiquitin (#3936) and Normal Rabbit IgG (#2729) were all from Cell Signaling Technology (Danvers, MA, USA).

4-Nitroquinoline 1-oxide (4-NQO) and Capzimin were purchased from Sigma-Aldrich. Thiolutin (THL) and Cycloheximide (CHX) were obtained from Tocris Bioscience (Avonmouth, Bristol, United Kingdom). MG132 was acquired from Selleck (Shanghai, China).

### Transfection and transduction

Small interfering RNAs (siRNAs) targeting PSMD14 and negative control were purchased from RIBOBIO (Guangzhou, China). Wild-type (WT) PSMD14 and mutants (the ΔJAMM, C120S and H113Q mutant) were cloned into pLVX-IRES-puro vector. Lentivirus containing SNAIL and the control were obtained from Genechem (Shanghai, China). Transfection and transduction were performed as previously described 30.

### Transwell assay

For invasion or migration assay *in vitro*, ESCC cells (KYSE 30, 3✕10^4^ cells; KYSE 150, 1✕10^5^ cells) were planted into transwell inserts precoated with Matrigel (BD, Franklin Lakes, NJ, USA) or not. The bottom chambers were filled with 600 μl medium supplemented with 20% FBS. The migratory or invasive cells were fixed with methanol and stained with 0.1% crystal violet after incubation at 37 °C for 24 h. Cells were photographed and counted using an inverted microscope in three randomly selected fields.

### The wound healing assay

KYSE 30 or KYSE 150 cells were seeded into 6-well plates. Until the cells grew nearly to 100% confluence, we made scratches by using 10 μl pipette tips. Then, the cells were washed and cultured in fresh medium without FBS for another 24 h. Each image for the gaps were collected at the start (0 h) and endpoint (24 h) of the experiment.

### Cell spheroid assay

500 ESCC cells were seeded into 6-well ultra-low cluster plates and were cultured in DMEM/F12 serum-free medium supplemented with 2% B27 (Invitrogen), 2×10^-5^ mg/ml EGF (PeproTech, Rocky Hill, NJ, USA), 2×10^-5^ mg/ml bFGF (PeproTech), 0.4% BSA and 5×10^-3^ mg/ml insulin for about two weeks. The spheroids whose diameter were more than 75 μm were counted by using an inverted microscope (DMI6000B, Leica).

### Clonogenicity assay

Clonogenicity assay serves as a useful tool to test whether a given cancer therapy could impair the survival of tumor cells. KYSE 30 or KYSE 150 cells (500-1000 cells/well) were seeded in 2 mL medium with 10% FBS in a 6-well plate overnight. The cells were exposed to cisplatin (1×10^-2^ mM) in the presence or absence of THL for 2 h, and then cultured with fresh complete medium for 2 weeks. The cells were washed, fixed and then stained with 0.1% crystal violet. Colonies were photographed and the ones with more than 50 cells were counted under an inverted microscope.

### Flow cytometry

Flow cytometry was performed to analyze apoptosis rate. Treated cells were digested and resuspended as a single-cell suspension and then washed twice in PBS. Apoptosis was measured on the same FACS Canto II (BD) according to the manufacturer instruction of Annexin V-FITC/PI Apoptosis Detection kit (BD) and Annexin V-APC/7-AAD Apoptosis Detection kit (BioLegend, San Diego, CA, USA).

### Cell viability assay

KYSE 30 or KYSE 150 cells were seeded into 96-well plates at a density of 5000 cells per well and incubated overnight for stabilization. Then cells were treated with THL (1×10^-4^, 5×10^-4^, 1×10^-3^, 2×10^-3^, 4×10^-3^, 6×10^-3^, 8×10^-3^, 1×10^-2^ mM) or DMSO for 24 h. An MTT assay was used to measure cell viability. A total volume of 20 μL of MTT was added for 4 h at 37 °C in dark. The MTT crystal was dissolved in DMSO, and the absorbance (490 nm) was measured using a microplate reader (Model 680, Bio-Rad Laboratories, Hercules, CA, USA). The half maximal inhibitory concentration (IC_50_) was calculated by using GraphPad Prism 6 (La Jolla, USA).

### Quantitative real-time PCR

Total RNA was extracted from cells using TRIzol reagent (Invitrogen, Waltham, MA, USA) according to standard instructions. PrimeScript™ RT Master Mix (TaKaRa, Shiga, Japan) was used to transcribe RNA to cDNA. Quantitative Real-time PCR (qPCR) was carried out using SYBR Premix Ex Taq^TM^ II (TaKaRa) on 7500 Real-Time PCR system (Applied Biosystems, Foster City, CA, USA). GAPDH was served as a loading control and the 2^-ΔΔCt^ method was used to evaluate the relative abundance of genes. The following primers were used in real-time PCR assay:

5' - AGGGCTGCTTTTAACTCTG - 3' (GAPDH, Forward), 5' - CTGGAAGATGGTGATGGG - 3' (GAPDH, Reverse), 5' - GGAGGAGGTATGCCTGGACT - 3' (PSMD14, Forward), 5' - TTAACAGTGCCAGGGAAGAGA - 3' (PSMD14, Reverse), 5' - TCGGAAGCCTAACTACAGCGA - 3' (SNAIL, Forward), 5' - AGATGAGCATTGGCAGCGAG - 3' (SNAIL, Reverse), 5' - AGCCAACCTTAACTGAGGAGT - 3' (CDH2, Forward), 5' - GGCAAGTTGATTGGAGGGATG - 3' (CDH2, Reverse), 5' - AGTCCACTGAGTACCGGAGAC - 3' (Vimentin, Forward), 5' - CATTTCACGCATCTGGCGTTC - 3' (Vimentin, Reverse).

### Protein extraction and immunoblotting

After treatment, cells were lysed in RIPA buffer with protease and phosphatase inhibitors (APExBIO, Houston, Texas, USA). The proteins (20-30 μg) were boiled for 5 min and separated in 10% SDS-PAGE gels and then transferred onto PVDF membranes (Merck Millipore, Billerica, MA, USA). The membranes were blocked in 5% non-fat milk and then incubated with primary antibodies overnight at 4 °C. After incubation with secondary antibody, the membranes were visualized with an enhanced chemiluminescence system kit (Cell Signaling Technology).

### Immunohistochemistry and Immunofluorescence staining

For immunohistochemistry (IHC) staining, paraffin embedded ESCC tissue samples were deparaffinized and rehydrated. Endogenous peroxidase was blocked by 3% H_2_O_2_, then citrate-based antigen retrieval was conducted. The samples were incubated with primary antibodies overnight at 4 °C. After incubation with HRP-conjugated secondary antibody, the sections were stained using DAB Substrate kit according to the manufacture's instruction.

For immunofluorescence (IF) staining, KYSE 30 and KYSE 150 cells were planted on 18-mm cover glasses overnight at 37 °C for stabilization, and then treated with THL or DMSO for 24 h. The cells were fixed and then permeabilized with Triton X-100. After blocked by 5% bovine serum albumin, the cells were incubated with primary antibodies at 4 °C overnight. The proteins were visualized by incubation with secondary antibody conjugated to Alexa Fluor 488 or Alexa Fluor 594 (Cell Signaling Technology) for 1 h at room temperature, and the nuclei were stained with DAPI (Thermo Fisher Scientific, Waltham, MA, USA) for another 10 min. The images were obtained using LSM 880 laser scanning confocal microscope (Zeiss, Oberkochen, Germany).

### Immunoprecipitation assay

The whole cell lysates were incubated with primary antibodies against PSMD14/SNAIL or normal IgG at 4 °C overnight, and 30 μl protein A/G agarose beads (Abmart, Shanghai, China) for another 2 h. The beads were washed for three times with ice-cold cell lysis buffer and the immunoprecipitated proteins were analyzed by immunoblotting.

### The *in vivo* ubiquitination assay

To detect the ubiquitination level of SNAIL, KYSE 30 and KYSE 150 cells exposed to THL were incubated with/without 1×10^-2^ mM MG132 for 8 h before they were harvested. The anti-SNAIL antibody was added into the supernatant to isolate ubiquitinated SNAIL. The specific procedure was performed as described in the immunoprecipitation assay, except for the detection of endogenous ubiquitin chains on SNAIL.

### TUNEL assay

The apoptotic ratio in ESCC cells and tissues was detected through TUNEL assay using a cell death kit (Beyotime, Shanghai, China). The experiment was accomplished following the manufacturers' protocol. Each slide was visualized using LSM 880 laser scanning confocal microscope (Zeiss).

### Animal experiments

All animal experimental protocols were approved by the Institutional Animal Care and Use Committee of Tianjin Medical University Cancer Institute and Hospital. For 4-NQO-induced ESCC animal model, 6-week-old male mice (BALB/c nude, HFK Bioscience, Beijing, China) were fed with water containing 4-NQO (5×10^-2^ mg/mL) for 16 weeks and then given with normal drinking water for another 12 weeks. Esophagi from the subjects were harvested at 28 weeks for further histopathological analysis.

To establish tumor xenograft model, 2✕10^6^ KYSE 30 cells were implanted subcutaneously into 6-week-old male nude mice. Until subcutaneous tumor establishment (>100 mm^3^), the mice were administered with saline, THL (0.75 mg/kg), cisplatin (CDDP, 2.5 mg/kg) or CDDP (2.5 mg/kg) plus THL (0.75 mg/kg) every 3 days. Tumor volume and body weight were measured every three days. After 21 days, all mice were sacrificed by cervical dislocation under anesthesia and the tumors were collected for further IHC detection.

### Statistical analysis

All assays except IHC and animal experiments were repeated three times at least. The statistical analyses were performed using GraphPad Prism 6. Data were presented as mean ± standard deviation (SD) and analyzed by using Student's* t*-test. The difference for which *P* < 0.05 was considered statistically significant.

## Results

### PSMD14 may be involved in ESCC tumorigenesis and its overexpression predicts poor prognosis

**Figure 1:**
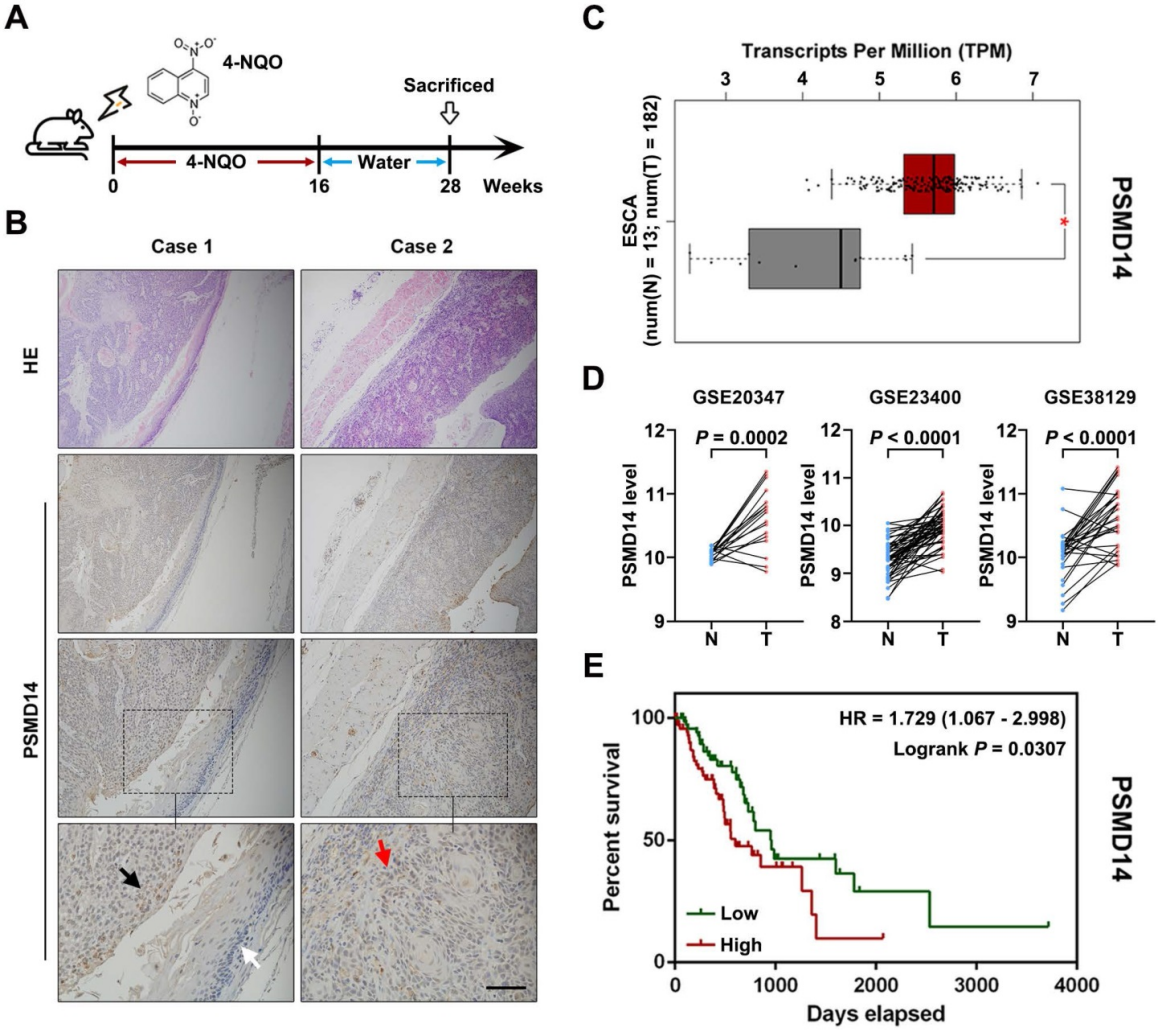
** PSMD14 overexpression predicts poor survival outcome in esophageal cancer. (A)** A murine ESCC model was established using chemical carcinogen 4-NQO (0.05 mg/mL) dissolved in drinking water. After 28 weeks, the esophagus tissues were harvested. **(B)** The images of H&E and immunohistochemical staining to assess the level of PSMD14 in normal esophageal epithelium (white arrows), dysplasia (black arrows) and ESCC (red arrows). Scale bar, 100 μm.** (C)** The analysis of TCGA database indicated that the levels of PSMD14 was elevated in ESCA (esophageal cancer). N, normal tissues. T, tumor samples. *, fold change ≥ 2 and *P <* 0.05.** (D)** Three independent cohorts in GEO database (GSE20347, GSE23400 and GSE38129) were analyzed to assess the level of PSMD14 in ESCC. N, normal tissues. T, tumor samples.** (E)** Kaplan-Meier survival curve showed that PSMD14 overexpression indicated poor prognosis in ESCA. HR, hazard rate.

To begin with, we explored PSMD14 expression in ESCC tumorigenesis using a 4-NQO-induced ESCC model (Figure 1A). As shown in Figure 1B, the expression of PSMD14 was enhanced from normal epithelial tissue to dysplasia and ESCC, which suggested that PSMD14 may be indispensable during the occurrence of ESCC. Then, we reconfirmed PSMD14 expression through in silico study of public databases. The analysis of esophageal cancer (EC) dataset in TCGA (The Cancer Genome Atlas) database by using GEPIA online software (gepia.cancer-pku.cn) demonstrated that compared with normal esophageal tissues, PSMD14 was overexpressed in esophageal cancer (Figure 1C). In addition, we analyzed three ESCC cohorts from GEO (Gene Expression Omnibus) database and found that the level of PSMD14 in ESCC samples was significantly higher than that in normal esophageal tissues (Figure 1D). Besides, EC patients with PSMD14 overexpression had a shorter overall survival (Figure 1E, HR = 1.729, *P* = 0.0307). Interestingly, other proteasome subunits were also expressed highly in EC (Figure S1) and the elevated expression of PSMA2, PSMA5, PSMC6, PSMD5, PSMD10 indicated poor prognosis of EC patients respectively (Figure S2), which revealed a crucial role of 26S proteasome in esophageal cancer. These results suggest that PSMD14 could be a novel biomarker for early diagnosis and prognosis prediction in ESCC.

### THL induces apoptosis of ESCC cells via suppressing DUB activity of PSMD14

**Figure 2:**
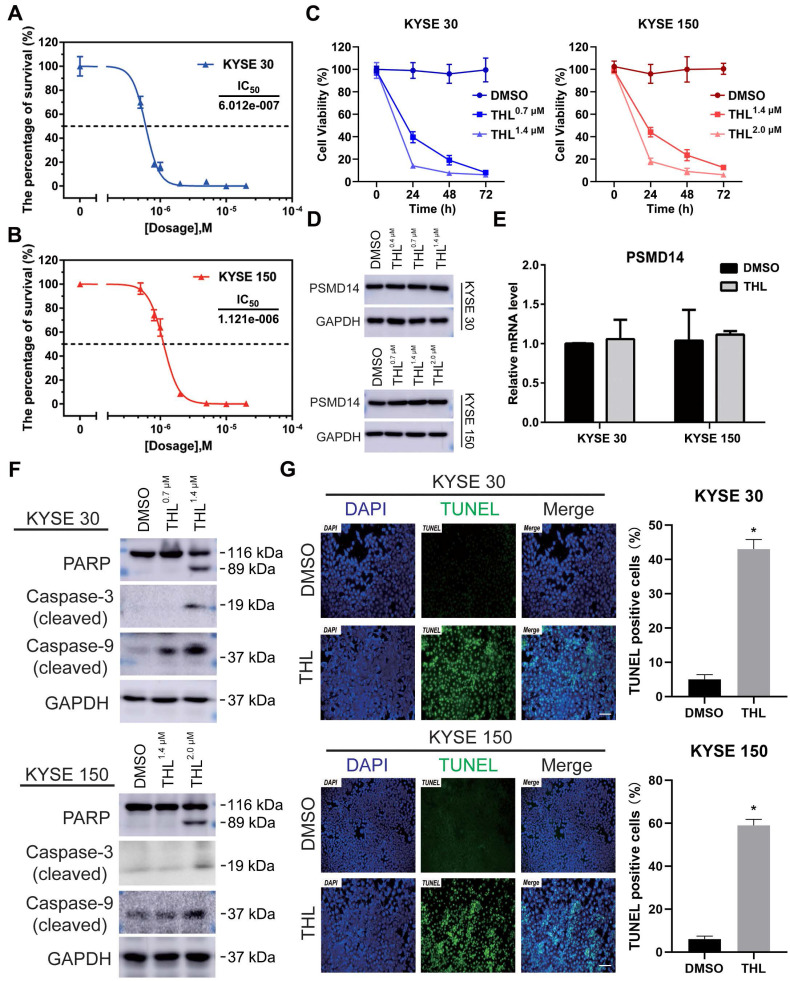
** THL suppresses DUB activity of PSMD14 and induces apoptosis of ESCC cells *in vitro*. (A-B)** MTT assay was performed to calculate the IC_50_ of THL in KYSE 30 **(A)** and KYSE 150 cell lines **(B)** for 24 h. **(C)** The cell proliferation of KYSE 30 and KYSE 150 cells was significantly inhibited after THL treatment. Cells were treated with THL or DMSO for 1, 2, 3 days respectively, then cell viability was measured by using MTT assay. **(D)** The results of immunoblotting showed that THL had little effect on the protein level of PSMD14. **(E)** The mRNA level of PSMD14 was analyzed by qPCR assay after the treatment of THL for 24 h. **(F)** The expressions of apoptosis-related proteins were analyzed by immunoblotting, including PARP, cleaved Caspase-3 and cleaved Caspase-9. **(G)** THL (1.4×10^-3^ mM in KYSE 30 cells and 2.0×10^-3^ mM in KYSE 150 cells) contributed to a considerable increase in the number of TUNEL positive cells, suggesting that THL stimulated cell apoptosis in ESCC. Scale bar, 100 μm. Data in this figure, mean ± SD, *P < 0.05.

**Figure 3:**
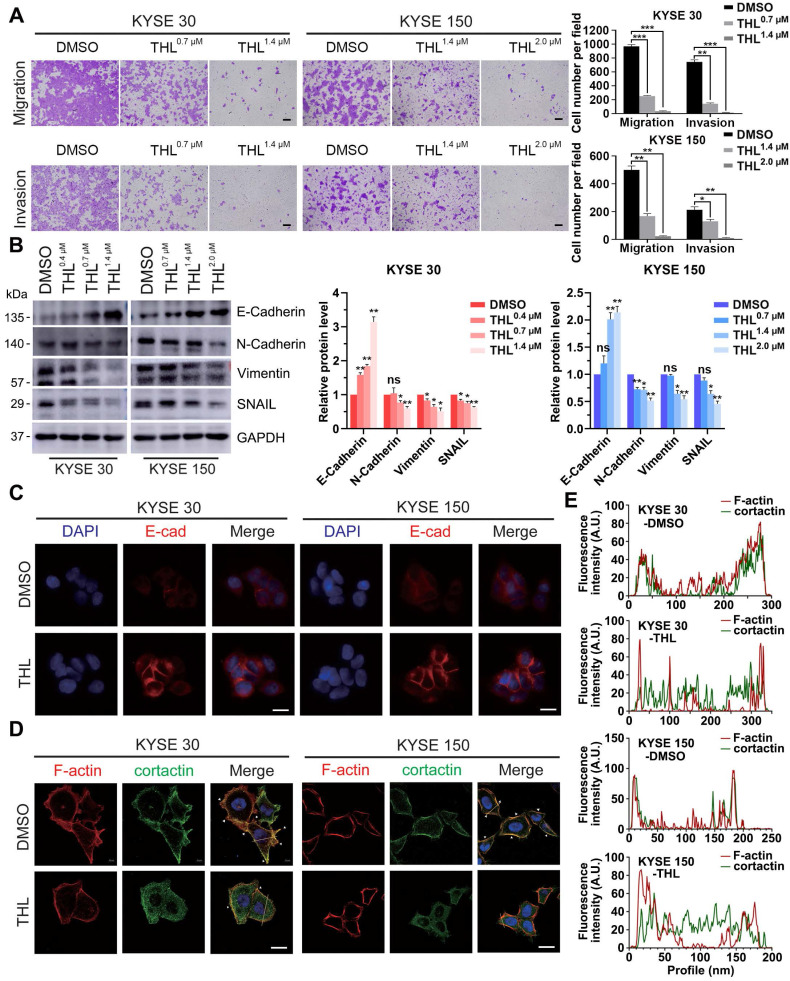
** THL undermines cell motility and reverses EMT process. (A)** Transwell assay was performed to show that THL attenuated significantly migration and invasion abilities of KYSE 30 and KYSE 150 cells. Scale bars, 100 μm. **(B)** The proteins of E-Cadherin, N-Cadherin, Vimentin and SNAIL were determined by immunoblotting in indicated treated ESCC cells. **(C)** The level of E-Cadherin was assessed by immunofluorescence in KYSE 30 and KYSE 150 cells treated with THL or DMSO for 24 h. Scale bar, 20 μm. **(D)** Immunofluorescence of F-actin and cortactin in DMSO and THL-treated cells. Scale bar, 20 μm. **(E)** The Fluorescence intensities of F-actin and cortactin along with the yellow lines marked in **(D)**. Data in this figure, mean ± SD, *P < 0.05, **P < 0.01, ***P < 0.001.

To investigate the effect of THL (Figure S3A) on PSMD14, we firstly performed Ubiquitin-AMC assay and found that the DUB activity of PSMD14 was significantly inhibited by THL in a dose-dependent manner (Figure S3B). Of note, the activities of other DUBs belonging to JAMM family were not affected efficiently by THL (Figure S3C), which provided evidence for the specificity of THL against PSMD14. Interestingly, the suppression of THL on* S. cerevisiae* Rpn11-Rpn8 heterodimer was weaker than that on PSMD14 (Figure 3D). It is possible that free AMC was still produced by Rpn8, which is also a DUB, even though Rpn11 was inhibited by THL. Then, we examined the protein expression of PSMD14 in a panel of ESCC cell lines. The results indicated that KYSE 30 and KYSE 150 cell lines showed higher levels of PSMD14 than other cell lines (Figure S4), therefore, we selected KYSE 30 and KYSE 150 cells for further experiments. After measuring the IC_50_ of THL in KYSE 30 (IC_50_ = 6.012×10^-7^ mol/L) and KYSE 150 (IC_50_ = 1.121×10^-6^ mol/L) cells (Figure 2A and 2B), we found that THL could obviously impair cell viability in ESCC (Figure 2C). Interestingly, no difference in PSMD14 protein expression in THL-treated cells was observed (Figure 2D). Meanwhile, we observed that transcriptional factor E2F1, a target of PSMD14, was inhibited by THL (Figure S5). Besides, the mRNA level of PSMD14 was not affected significantly in both ESCC cell lines tested (Figure 2E), indicating that THL only inhibited the DUB activity of PSMD14.

We next investigated the effect of THL on cell viability of ESCC cells *in vitro*. Immunoblotting assay was used to evaluate the level of apoptosis-related proteins. Elevated expression of cleaved PARP is considered as an important indicator of cell apoptosis. As shown in Figure 2F, THL treatment promoted the cleavage of PARP as well as Caspase-3 and Caspase-9. Furthermore, we employed a TUNEL assay to examine DNA fragmentation, the hallmark of apoptotic cells, in ESCC cells. The IF images demonstrated that the percentage of TUNEL positive cells was dramatically increased under the treatment of THL (Figure 2G). Similarly, PSMD14 knockdown led to apoptosis in both KYSE 30 and KYSE 150 cells (Figure S6). Together, these results above suggest that THL stimulates apoptosis of ESCC cells *in vitro* by attenuating PSMD14 DUB activity.

### THL retards motility of ESCC cells *in vitro* and reverses EMT process

Metastasis is one of major contributors to poor prognosis in ESCC. Therefore, we further investigated the influence of THL on cell motility in ESCC. Results from the transwell assays demonstrated that the migration and invasion of KYSE 30 and KYSE 150 cells were significantly retarded by THL in a dose-dependent manner (Figure 3A). And we also verified that wound healing was delayed in both ESCC cell lines treated with THL (Figure S7).

Of note, EMT is widely regarded as one of the most critical causes of metastasis in multiple cancers. The decrease of E-Cadherin protein was regarded as the initiation of EMT. Thus, we assessed the expression of E-Cadherin and other EMT markers, such as N-Cadherin, Vimentin and SNAIL, in ESCC cells under THL treatment. In KYSE 30 and KYSE 150 cells, THL significantly increased the expression of E-Cadherin and suppressed N-Cadherin and Vimentin in a dose-dependent manner (Figure 3B). The results of IF and qPCR shown in Figure 3C and Figure S8 were in line with the results shown above. Compared with other EMT transcription factors, THL dramatically decreased the expression of SNAIL (Figure 3B and Figure S9), which regulates gene transcription and triggers EMT. These results indicated that THL could reverse EMT process. Moreover, the effect of THL on the cytoskeleton of ESCC cells was analyzed. When ESCC cells were exposed to THL, the co-localization between F-actin and cortactin along the cellular membrane (Figure 3D, white arrows), which promotes the formation of invadopodia, was notably decreased (Figure 3D-E). Collectively, these data above suggest that THL could dramatically weaken the invasion and migration capacities of ESCC cells *in vitro* by interrupting EMT process.

### THL impairs cell stemness and sensitizes ESCC cells to CDDP *in vitro*

**Figure 4:**
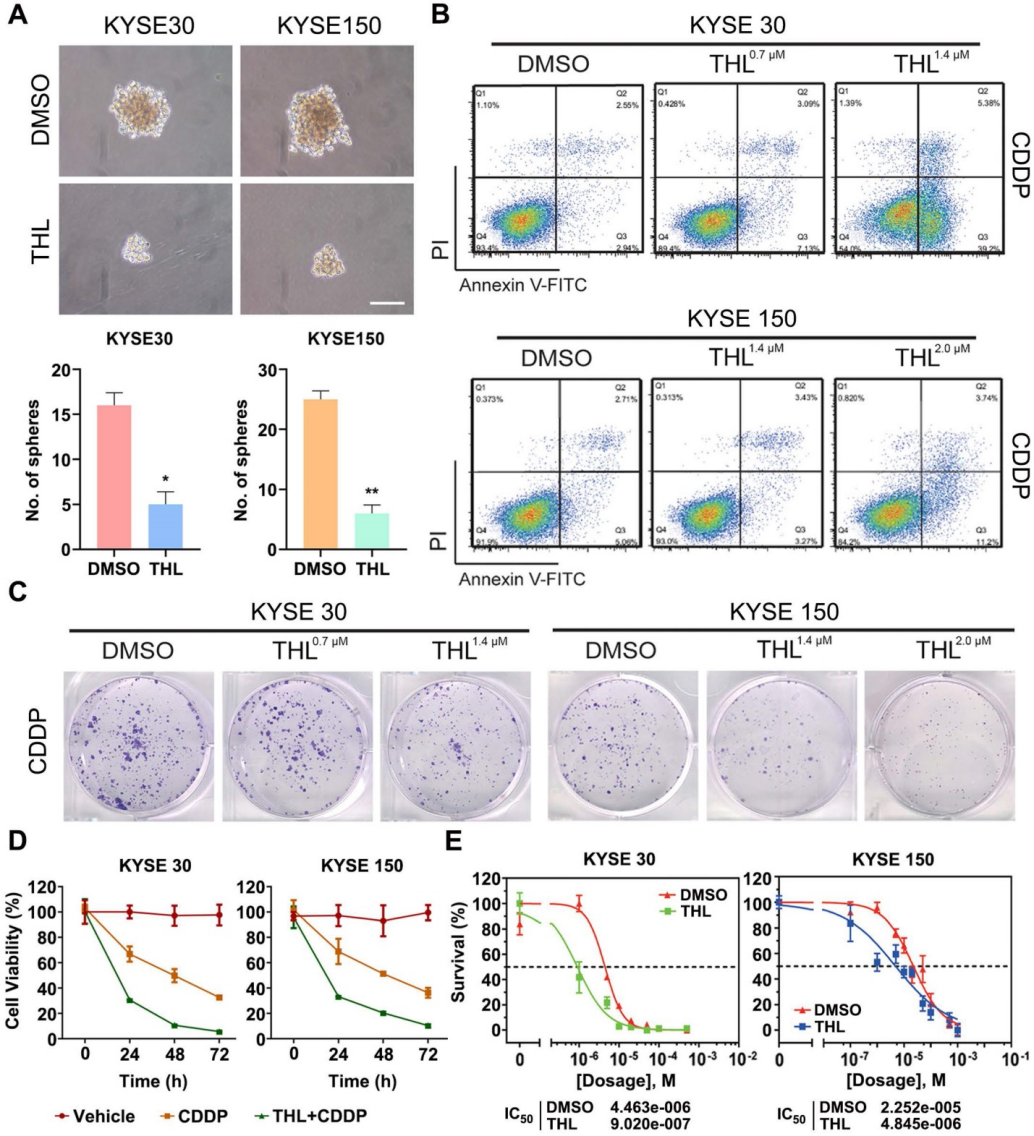
** THL impairs cell stemness and sensitizes ESCC cells to CDDP* in vitro.* (A)** Cell spheroid assay was performed to detect the effect of THL on ESCC cell stemness. **(B)** Cell apoptosis of KYSE 30 was measured by flow cytometry under indicated treatment. **(C)** Representative images showed reduction of colony formation ability in KYSE 30 cells pretreated with CDDP plus THL. **(D)** ESCC cells were treated with CDDP or CDDP combined with THL for 24, 48, 72 h, respectively, and then cell viability was measured by MTT assay. **(E)** THL-pretreated ESCC cells showed a lower IC_50_ of CDDP compared to DMSO groups. Data in this figure, mean ± SD, *P < 0.05, **P < 0.01.

Previous studies declare that EMT confers tumor stemness and chemoresistance 17, 31, 32. THL was verified to reverse EMT above; therefore, we analyzed the potential effect of THL on cell stemness and response of ESCC cells to CDDP, the standard chemotherapy regimen for ESCC. As shown in Figure 4A, the size and number of spheroids were significantly decreased in THL-pretreated ESCC cells, suggesting that THL could weaken cell stemness in ESCC. Pre-treated with THL for 2 h, ESCC cells were exposed to CDDP for another 24 h. Then, we observed that the apoptosis rate was significantly increased in both two cell lines treated as shown by flow cytometry (Figure 4B). Additionally, both the size and number of colonies were reduced in KYSE 30 and KYSE 150 cells pre-treated with CDDP plus THL for 2 h (Figure 4C). And the viability of ESCC cells was impaired dramatically in the combined treatment group compared to CDDP group (Figure 4D). MTT assay results demonstrated that pre-treatment of THL could lower the IC_50_ of CDDP to 9.02×10^-7^ mol/L in KYSE 30 cells and to 4.845×10^-6^ mol/L in KYSE 150 cells respectively (Figure 4E), indicating that THL improved the sensitivity to CDDP in ESCC cells *in vitro*.

In addition to THL, the effect of another well-known PSMD14 specific inhibitor Capzimin in ESCC was assessed to make comparison between them. Compared with THL, Capzimin induced cell death in ESCC with a higher IC_50_ (Figure S10A). Treated with Capzimin, the viability and motility of ESCC cells were obviously inhibited (Figure S10B and S10C), and the protein expression of SNAIL was reduced in both KYSE 30 and KYSE 150 cells (Figure S10D). However, the chemo-sensitizing effect of Capzimin was not obvious in relative to THL (not shown). These results indicate that THL may be a rational and promising PSMD14 inhibitor in ESCC rather than Capzimin.

### THL weakens the interaction between PSMD14 and SNAIL to accelerate the degradation of SNAIL

**Figure 5:**
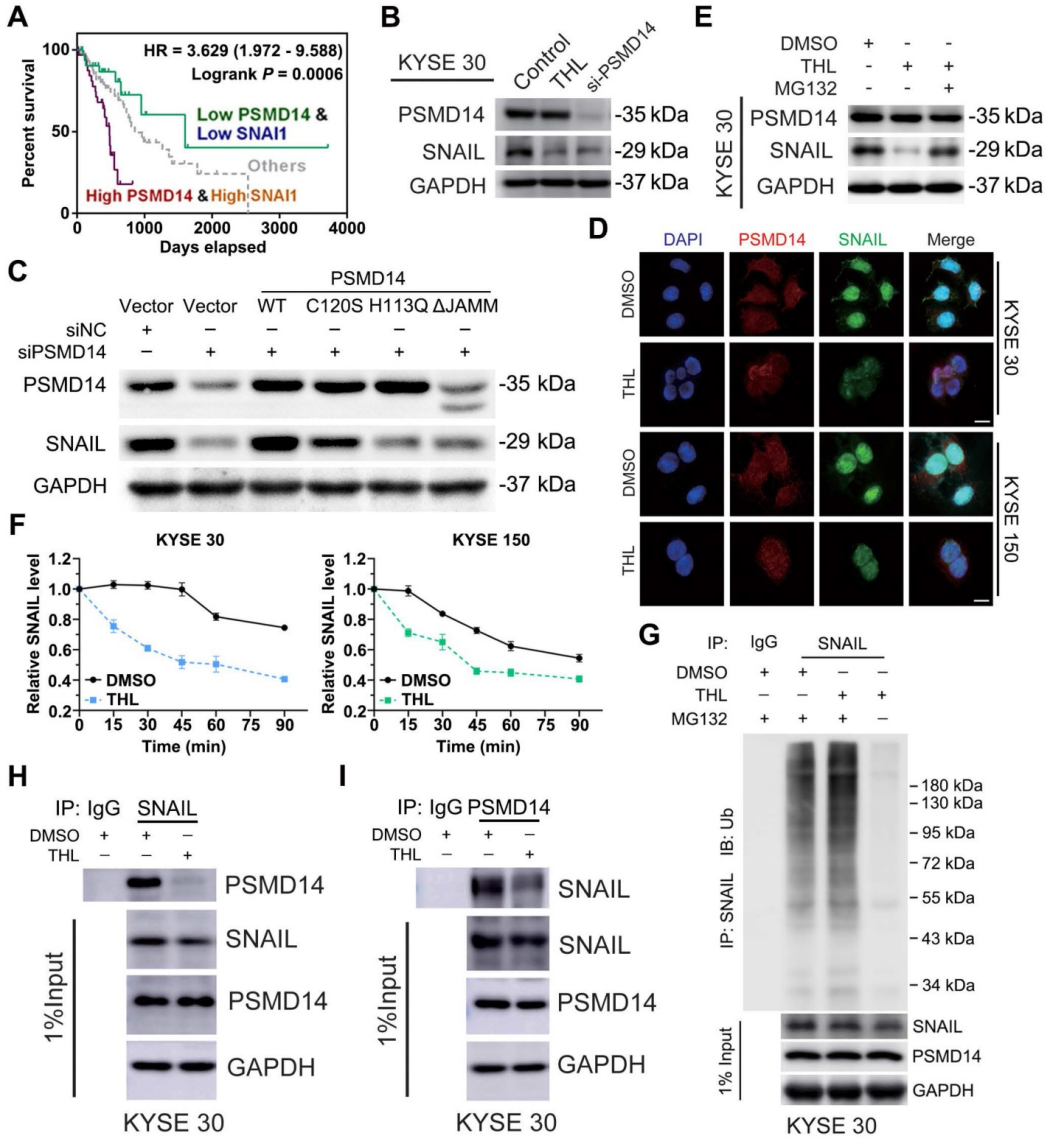
** THL accelerates the degradation of SNAIL through ubiquitin proteasome system. (A)** High concomitant expression of PSMD14/SNAIL predicted shorter overall survival in esophageal cancer. **(B)** The results of immunoblotting showed that both THL and si-PSMD14 inhibited the protein level of SNAIL in KYSE 30 cells. (C) KYSE150 cells were transfected with siNC or siPSMD14 together with wild-type or mutant PSMD14. The cells were collected 48 h after transfection and immunoblotted with the indicated antibodies. **(D)** Immunofluorescence of PSMD14 and SNAIL in KYSE 30 and KYSE 150 cells after THL treatment for 24 h compared with DMSO. Scale bar, 20 μm. **(E)** The protein levels of PSMD14 and SNAIL were examined by immunoblotting in KYSE 30 cells with indicated treatments. **(F)** KYSE 30 and KYSE 150 cells, pretreated with THL or DMSO for 24 h, were exposed to 0.05 mg/ml CHX at the indicated time point for 0, 15, 30, 45, 60, 90 min. The SNAIL protein expression was analyzed by immunoblotting and quantified by ImageJ software. **(G)** Pretreated with THL or DMSO for 24 h, KYSE 30 cells were incubated with MG132 for another 12 h. The endogenous SNAIL was immunoprecipitated using anti-SNAIL antibody and its poly-ubiquitination level was measured by anti-ubiquitin antibody. 1% input of cell lysates was used to analyze protein levels of PSMD14 and SNAIL. **(H)** KYSE 30 cells were treated with THL or DMSO for 24 h. Cell lysates were immunoprecipitated to pull down PSMD14 by anti-SNAIL antibody. **(I)** Anti-PSMD14 antibody was used to pull down SNAIL by a co-immunoprecipitation assay, as described in **(H)**. Data in this figure, mean ± SD. SNAI1, an alias for SNAIL.

After validating anti-tumor activity of THL in ESCC, we aspired to delineate the underlying mechanism controlled by THL. In view of the critical effect of SNAIL-mediated EMT on metastasis and chemoresistance, we concentrated on illustrating how THL regulated SNAIL expression. TCGA database analysis showed that SNAIL was elevated in esophageal cancer relative to normal tissues and indicated unfavorable prognosis (Figure S11A-B). Furthermore, pairwise correlation analysis showed a statistically significant positive correlation between PSMD14 and SNAIL in terms of transcripts per million (Figure S11C, *P* = 0.044). As shown in Figure S11D, we identified SNAIL target genes as an enriched pathway in the tumor specimens with high abundancy of PSMD14 from three ESCC cohorts by using Gene set enrichment analysis (GSEA). Meanwhile, the protein expression of PSMD14 correlated positively with that of SNAIL among these ESCC cell lines (Figure S4), reconfirming the positive relationship between them, which is similar to the study reported by Zhu *et al.* 33. Interestingly, further stratification of patient groups based on high PSMD14/SNAIL expression improved the predictive capability of either one (Figure 5A, HR = 3.629, *P* = 0.0006).

Then, we blocked PSMD14 protein as a positive control using a pool of siRNAs against PSMD14. As shown in Figure 5B and Figure S12A, THL and si-PSMD14 both dramatically inhibited SNAIL expression, but the mRNA level of SNAIL was not fluctuated significantly in KYSE 30 and KYSE 150 cells (Figure S12B). Recent studies demonstrated that other DUBs such as DUB3 and USP27X also deubiquitinated and stabilized SNAIL to promote tumor progression 19, 34, 35. However, THL did not significantly affect the activity of DUB3 (Figure S3E). Compared with wild-type PSMD14, restoration of PSMD14 expression with the enzyme-deficient mutants (C120S-, H113Q- and ΔJAMM-PSMD14) did not rescue the expression of SNAIL caused by the deletion of endogenous PSMD14 in KYSE 150 cells (Figure 5C). These data indicated that THL regulated protein expression of SNAIL through inhibiting the DUB activity of PSMD14. We also found that THL suppressed SNAIL level in a time-dependent manner (Figure S12C). Immunofluorescence staining showed no significant change in PSMD14 expression and localization, but SNAIL level in THL-treated cells and even that in the nucleus were much lower (Figure 5D).

As shown in Figure S12D, the proteasome inhibitor MG132 enhanced the expression of SNAIL, which confirmed that SNAIL degradation was mediated by ubiquitin-proteasome system. Besides, MG132 nearly abrogated the inhibition of THL and si-PSMD14 on SNAIL in both KYSE 30 and KYSE 150 cells (Figure 5E and Figure S12E-F), suggesting THL regulated proteasome-mediated degradation of SNAIL by targeting PSMD14. After the treatment of THL for 24 h, KYSE 30 and KYSE 150 cells were treated with CHX to block protein synthesis at the indicated time and then the effect of THL on SNAIL stability was detected. The results of immunoblotting assay showed that THL shortened the half-life of SNAIL and accelerated its degradation (Figure 5F and Figure S12G). Besides, we validated that the ubiquitination level of SNAIL was greatly elevated by THL (Figure 5G and Figure S12H). Furthermore, co-immunoprecipitation assay was conducted to test the impact of THL on the interaction between PSMD14 and SNAIL in ESCC cells. Compared with DMSO, THL conferred the decrease of PSMD14 co-immunoprecipitating with SNAIL (Figure 5H and Figure S12I). Meanwhile, the level of SNAIL which interacts with PSMD14 was also reduced when ESCC cells were exposed to THL (Figure 5I and Figure S12J). Taken together, these results indicate that THL blocks the DUB activity of PSMD14 to prevent it from interacting with SNAIL, resulting in the ubiquitination and instability of SNAIL.

### SNAIL hampers anti-tumor effect of THL on ESCC cells

**Figure 6:**
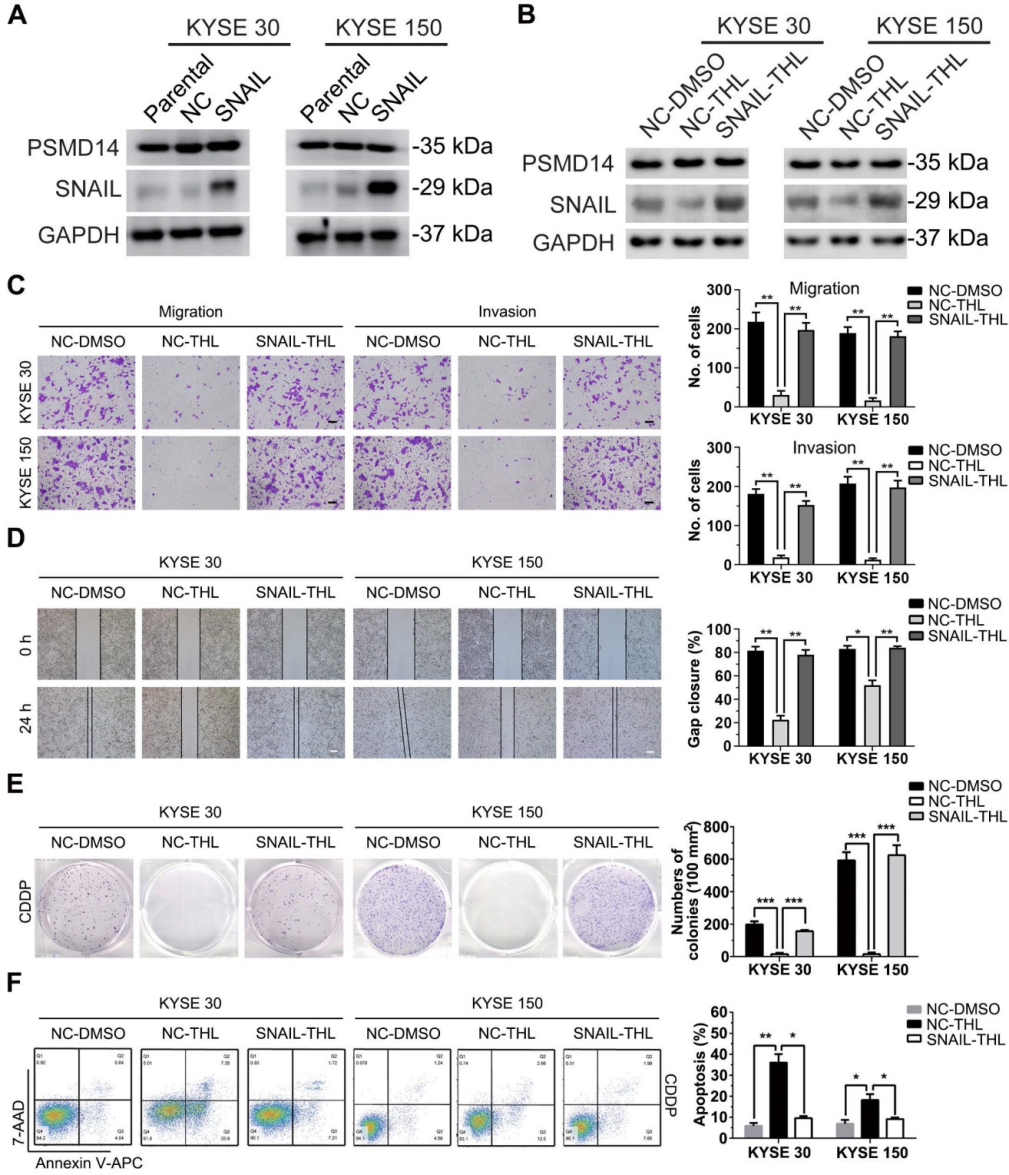
** SNAIL reduces the anti-tumor effect of THL on ESCC cells. (A)** The protein levels of PSMD14 and SNAIL was examined by immunoblotting in KYSE 30 and KYSE 150 cells stably expressing SNAIL or negative control. NC, negative control. **(B)** Immunoblotting assay was performed to measure the expressions of PSMD14 and SNAIL in the cells described in **(A)** under THL treatment. **(C-D)** SNAIL restored the THL-impaired motility capacity of ESCC cells, which was determined by using transwell assay **(C)** and wound healing assay** (D)**. Scale bar in** (C)**, 100 μm. Scale bar in** (D)**, 200 μm.** (E-F)** The results of clonogenicity assay **(E)** and flow cytometry **(F)** showed that overexpressed SNAIL increased CDDP resistance in KYSE 30 and KYSE 150 cells treated with THL. The concentration of THL was 1.4×10^-3^ mM in KYSE 30 cells and 2.0×10^-3^ mM in KYSE 150 cells, respectively. Data in this figure, mean ± SD, *P < 0.05, **P < 0.01, ***P < 0.001.

Subsequently, we established stable KYSE 30 and KYSE 150 clones expressing SNAIL respectively (Figure 6A) for further confirming that THL inhibited malignancy of ESCC cells through suppressing PSMD14/SNAIL axis. Compared to negative control, forced expression of SNAIL mitigated the THL-mediated suppression of SNAIL (Figure 6B). As expected, SNAIL re-expression rescued THL-impaired the capacities of motility and clonogenicity in KYSE 30 and KYSE 150 cells, respectively (Figure 6C-E). Moreover, the ESCC cells expressing SNAIL became much resistant to CDDP when pre-treat with THL (Figure 6F). In brief, these results above indicate that THL exerts anti-tumor effect in ESCC through PSMD14/SNAIL axis.

### THL reverses EMT and enhances ESCC sensitivity to CDDP *in vivo*

**Figure 7:**
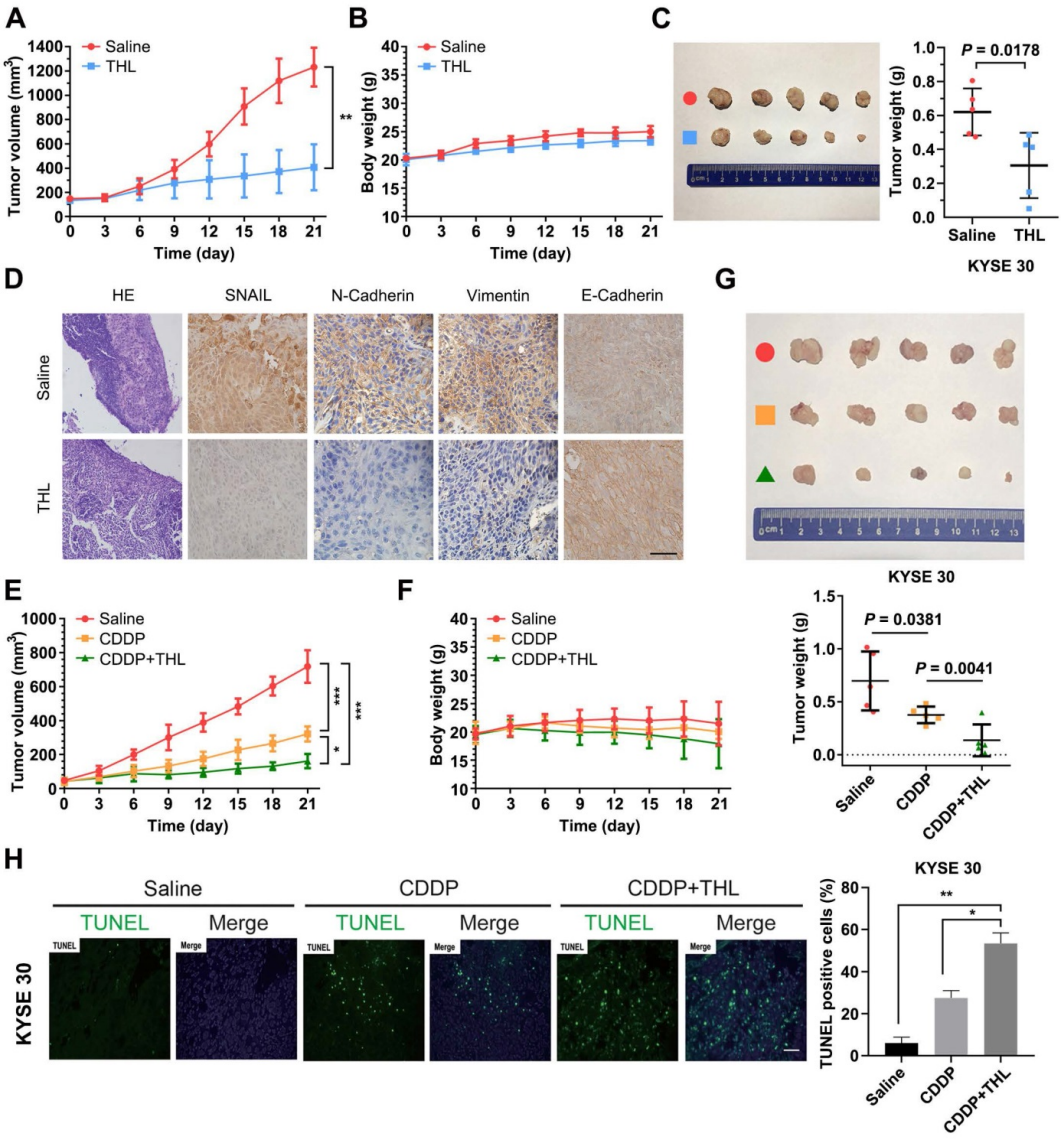
** THL slows ESCC tumor growth and improves sensitivity to CDDP *in vivo*. (A)** Tumor volume was measured every 3 days to determine the growth rate of THL-treated xenografts and DMSO-treated control. **(B)** No obvious difference in body weight was found between THL-treated group and negative control. **(C)** Representative images for xenografts of THL-treated or DMSO-treated mice. THL significantly inhibited the *in vivo* growth of KYSE 30 cells.** (D)** The images of H&E and immunohistochemical staining to assess the levels of SNAIL, N-Cadherin, Vimentin and E-Cadherin in xenografts treated with THL or DMSO. Scale bar, 100 μm. **(E)** The tumor volume of xenografts treated with saline, CDDP, CDDP combined with THL, respectively. **(F)** The body weight of animals with indicated treatment described in **(E)** was recorded every 3 days. **(G)** Representative images for indicated treated ESCC xenografts. The size and weight of tumors were significantly decreased in combined treatment group. **(H)** A TUNEL assay showed more induced apoptotic nuclei in combined treatment group than in CDDP treatment group. Scale bar, 100 μm. Data in this figure, mean ± SD, *P < 0.05, **P < 0.01, ***P < 0.001.

To assess the impact of THL on ESCC *in vivo*, KYSE 30 cells were used to establish subcutaneous xenograft model for further experiments. As shown in Figure 7A, the *in vivo* growth of KYSE 30 cells was significantly inhibited by THL, while no obvious difference in body weight was observed between two groups (Figure 7B). After 21 days, the volume and weight of tumor in THL-treated group were much less than that in saline-treated group (Figure 7C). Meanwhile, IHC staining showed enhanced expression of E-Cadherin and attenuated levels of SNAIL, N-Cadherin and Vimentin in xenografts with the administration of THL (Figure 7D), which suggested that THL reversed EMT process and may further abrogate the initiation of metastasis. In addition, we found that THL obviously improved the chemotherapeutic efficacy *in vivo* without significant loss of body weight (Figure 7E-G), due to the treatment with low dose of CDDP. The outcome of TUNEL assay indicated that combined CDDP and THL treatment strongly induced apoptosis of xenografts relative to other two groups (Figure 7H). In general, these data suggest that the regimen of CDDP combined with THL could be a promising therapeutic strategy for patients with ESCC, particularly the ones with no currently effective treatment options.

## Discussion

The specific components involved in aberrant signaling pathway were usually translated to act as therapeutic targets for revolutionizing therapy strategy in multiple cancers. As a candidate, PSMD14 is highly expressed in various cancers and performs as an oncogene to promote tumor development and progression 12-14. PSMD14 could remove the ubiquitin chain from targeted proteins at the entrance of 19S proteasome, facilitating proteins to translocate into the 20S proteasome subunit for further degradation 36, 37. On the contrary, previous studies revealed that as a DUB, PSMD14 could also suppress the degradation of specific proteins, such as E2F1 14, GRB2 38 and ALK2 receptor 39. Consequently, PSMD14 is speculated to possess a 'proofreading' function, like a “controller” of recycling station, to determine the fate of substrates. Here, we verified that PSMD14 was overexpressed in esophageal cancer and predicted poor prognosis by analyzing TCGA database, which is in line with the results stated by Zhu *et al.* 33. Furthermore, we found that PSMD14 may participate in the tumorigenesis of ESCC through a 4-NQO-induced ESCC model. Therefore, it seems to be a promising strategy to develop small-molecule inhibitors targeting PSMD14 for ESCC treatment.

Despite of the potential inhibition on other JAMM proteases at high concentration, THL could inhibit PSMD14 with a minimum IC_50_ 29. Through Ubiquitin-AMC assay, we found that the IC_50_ of THL on PSMD14 was much lower than that on other JAMM family DUBs, which provided evidence for the specificity of THL on PSMD14. In view of the pivotal role of PSMD14 in malignant tumors, THL is potentially interesting as an anti-tumor drug. The data of this study demonstrated that THL significantly suppressed DUB activity of PSMD14 in a dose-dependent manner, while neither the protein expression nor mRNA level of PSMD14 was affected. Therefore, THL exhibited potent anti-tumor activity in ESCC by impairing PSMD14 DUB function. However, the function and mechanism of THL remain to be further studied.

Further experiments results showed that THL dramatically undermined tumor cell motility and resistance to chemotherapy drugs, two major contributors to poor outcome of ESCC patients. Importantly, previous study affirmed that tumor cells become spindle-shaped and motile, and acquire a higher resistance to chemotherapy when they undergo an EMT process 18, 40. Therefore, the effect of THL on EMT was detected. The results of IF and immunoblotting revealed that THL treatment improved the expression of E-Cadherin but inhibited the mesenchymal markers including SNAIL, suggesting that THL could reverse EMT process. In the meanwhile, the images obtained by confocal microscope demonstrated that the fluorescence intensity of F-actin was not correlated positively with that of cortactin under the treatment of THL. So, we believed that THL affected cytoskeletal rearrangement and abrogated the formation of invadopodia, which is of great importance for cell motility 41, 42.

**Figure 8:**
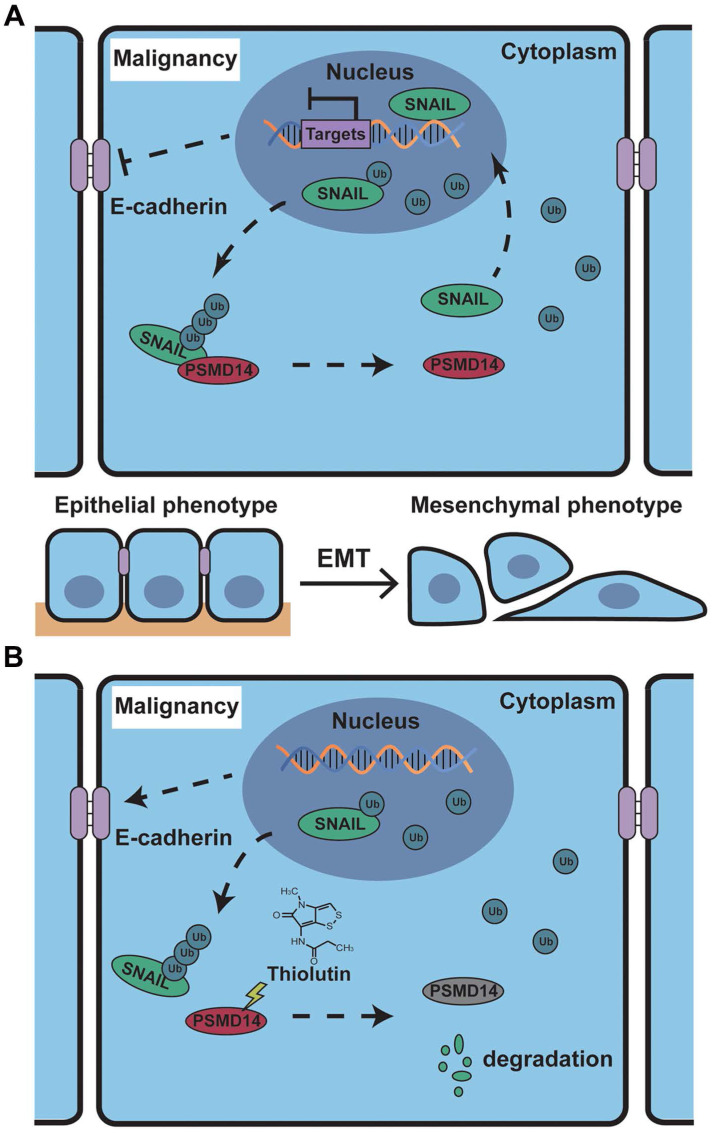
** Schematic of the mechanism of THL for suppressing ESCC malignancy. (A)** As a very unstable protein in normal cells, SNAIL is labeled by ubiquitin and then degraded by proteasome. However, in ESCC cells, the ubiquitin chains above are released by deubiquitinating enzyme PSMD14, which leads to the obstruction of SNAIL degradation. A large number of SNAIL gather in the nucleus to repress the transcription of E-Cadherin. As a critical molecule in the EMT process, the decrease of E-Cadherin triggers EMT to improve metastasis and chemoresistance in ESCC. **(B)** THL accelerates the proteasome-mediated degradation of SNAIL to reverse EMT process by attenuating the DUB activity of PSMD14.

It is well known that the transcription factor SNAIL represses E-Cadherin expression to trigger EMT 43, and functions as a critical oncogene in various cancers. But so far, no specific SNAIL-targeted drugs were approved by FDA. In ESCC, PSMD14 deubiquitinates and stabilizes SNAIL to promote tumor metastasis 33. Through in silico analysis, we identified SNAIL target genes as an enriched pathway in ESCC with high abundancy of PSMD14. Additionally, we analyzed TCGA database and found that high concomitant PSMD14/SNAIL expression indicated worse outcome of patients with esophageal cancer. Therefore, targeting PSMD14/SNAIL axis provides a novel challenge and opportunity to improve prognosis in ESCC. In this study, we found that THL, similar to si-PSMD14, could reduce the protein level of SNAIL, instead of SNAIL mRNA. Compared with the wild-type PSMD14, restoration of PSMD14 expression with enzyme-deficient mutants did not mitigate the inhibition of siPSMD14 on SNAIL protein, indicating that the DUB activity is crucial for the effect of PSMD14 on SNAIL. Then, we further assessed whether THL inhibited SNAIL through ubiquitin proteasome system (UPS). When the activity of proteasome was blocked, the protein level of SNAIL in the cells treated with THL was restored. Besides, THL reduced stabilization of SNAIL, which indicates that THL promotes UPS-mediated degradation of SNAIL. Moreover, we performed *in vivo* ubiquitination assay and found that THL greatly elevated the poly-ubiquitination of SNAIL, reconfirming that THL suppresses DUB activity of PSMD14. In addition, THL also prevented PSMD14 from interacting with SNAIL. These results verified that THL indeed suppressed SNAIL at the posttranslational level by blocking PSMD14 DUB activity. Further functional experiments revealed that SNAIL could partially compensate for the anti-tumor effect of THL. These findings validate that restriction of PSMD14-induced SNAIL accumulation is one of the major underlying mechanisms by which THL suppresses ESCC malignancy (Figure 8).

The results of *in vivo* experiments confirmed our* in vitro* results. The administration of THL inhibited tumor growth of ESCC xenograft. IHC analysis showed that the level of SNAIL in xenografts treated with THL was decreased whereas the expression of E-Cadherin on the cell membrane was increased, indicating the efficient inhibition of THL on EMT and even tumor metastasis. Furthermore, THL significantly enhanced xenografts sensitivity to CDDP and stimulated apoptosis *in vivo*, while no obvious change in body weight was observed. Therefore, THL may help improve response rate of chemotherapy and reduce side effects by decreasing the dosage of CDDP to benefit patients. In the current study, we introduced THL into the experimental animals by peritumoral injection, which might be the drawback of our *in vivo* experiments. Therefore, more *in vivo* tests of THL with several administration routes, such as intraperitoneal injection and oral administration, are warranted for further analysis.

It is worth noting that Lauinger L *et al.* demonstrated that THL could inhibit protein degradation by proteasome 29, which seems to be the opposite of what we found. Similarly, other inhibitors targeting PSMD14 are also reported to stabilize proteasome substrates and block proliferation of multiple myeloma (MM) cells 44-47, including those resistant to proteasome inhibitor which is an effective therapy for patients with MM 48, 49. Identification of PSMD14 inhibitor seems to be an alternative way to develop proteasome inhibition to treat cancer. However, in view of the two-sided role of PSMD14 in determining substrates fate, the inhibitor targeting PSMD14, which affects proteasome indirectly, could not be completely equated with proteasome inhibitor. The effect of PSMD14 inhibitors including THL should depend on the specific proteasome substrate. Therefore, there may exist no contradiction between the previous reports and our findings in this study. For another, proteasome inhibitors, such as bortezomib and carfilzomib, are not successful in the treatment of solid tumors 50, which may be more difficult to reach and distinguish less from normal tissues in their dependence on the ubiquitin proteasome system. On account of the *in vitro* and *in vivo* experiments results above, PSMD14 inhibitor, the one with higher targeting ability may be a safer and more effective candidate for tumor treatment.

In conclusion, this study reveals that THL, as a small-molecule inhibitor against PSMD14, attenuates migration and invasion and improves chemosensitivity of ESCC cells by suppressing PSMD14-induced SNAIL accumulation and EMT process. Our findings provide a rationale for THL's clinical applications in ESCC treatment.

## Supplementary Material

Supplementary figures.
