# Supplementary material for: The PSMD14 inhibitor Thiolutin as a novel therapeutic approach for esophageal squamous cell carcinoma through facilitating SNAIL degradation
Source: Theranostics. 2021 Apr 3;11(12):5847–62. doi: 10.7150/thno.46109 (PMC8058732; doi:10.7150/thno.46109)
Supplement: Supplementary file 1 — Supplementary figures. [file thnov11p5847s1.pdf]

Supplementary Figures

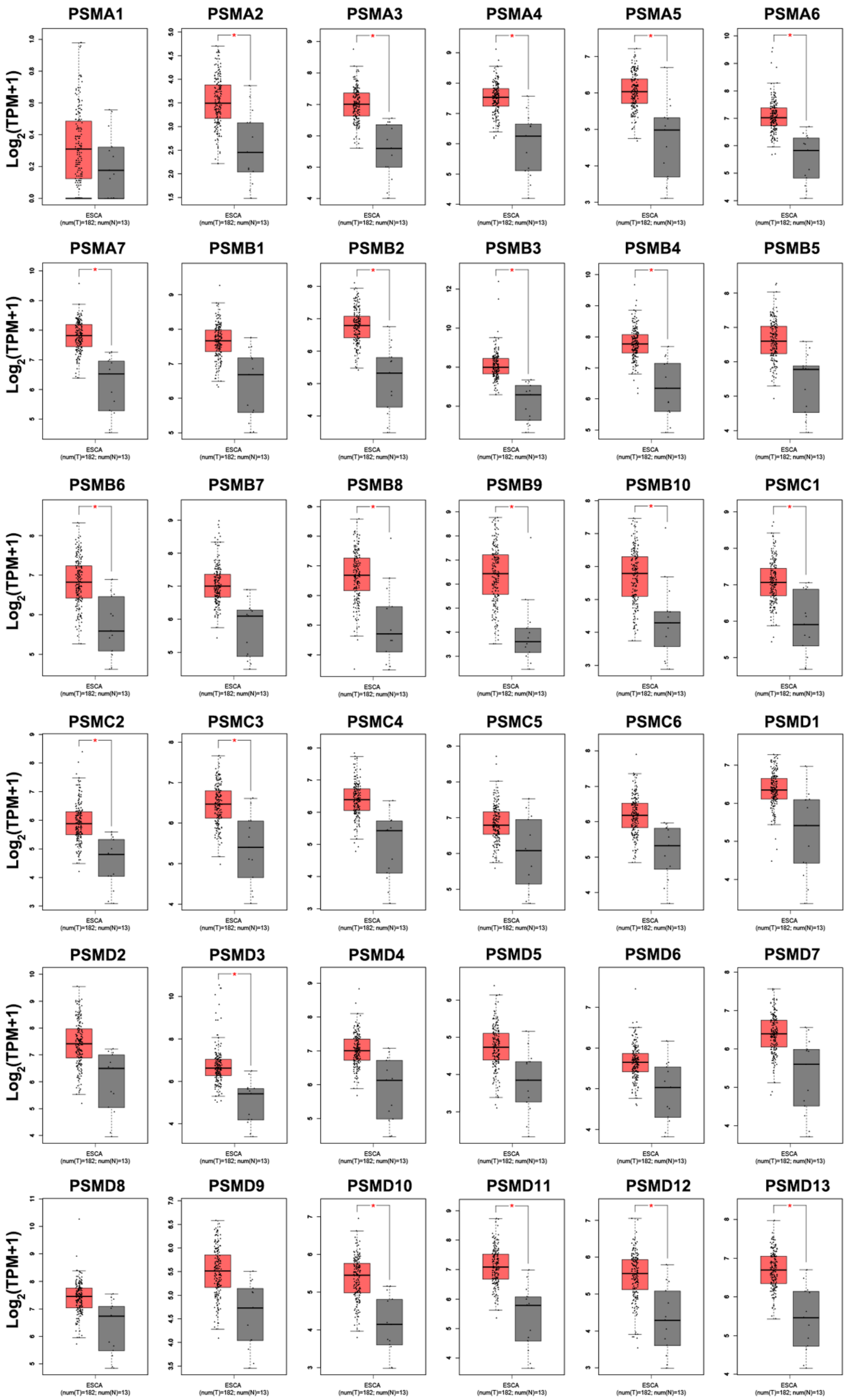

Figure S1. The expressions of 19S and 20S proteasome subunits in esophageal cancer specimens and normal esophageal tissues by analyzing TCGA database. TPM, transcripts per million. ESCA, esophageal cancer. N, normal tissues. T, tumor samples. *, fold change ≥ 2 and $P < 0.05$.

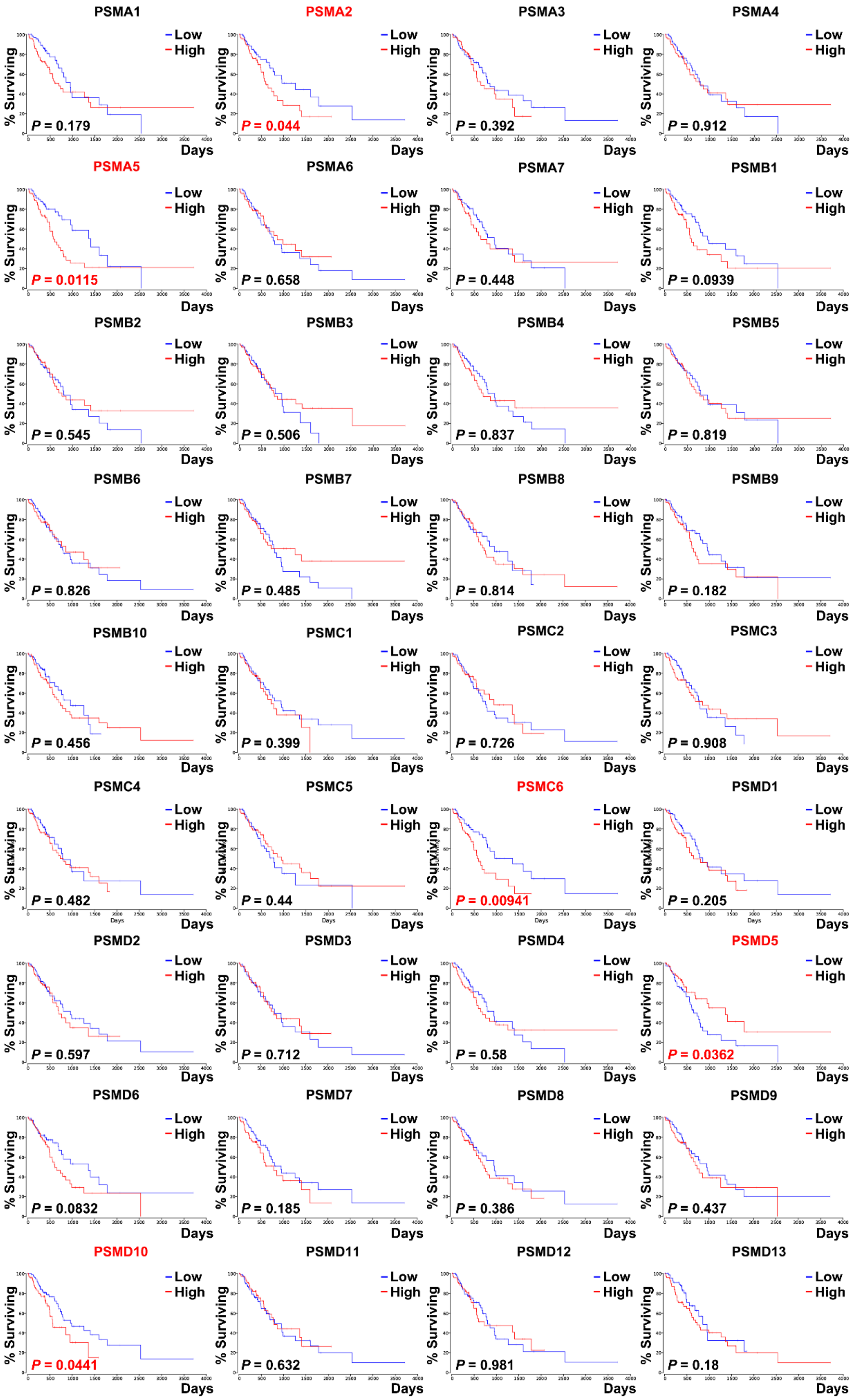

Figure S2. The Kaplan-Meier survival curves of ESCA patients based on the level of 19S

and 20S proteasome subunits. The aberrant expression of PSMA2, PSMA5, PSMC6, PSMD5, PSMD10 indicated poor prognosis in esophageal cancer, respectively.

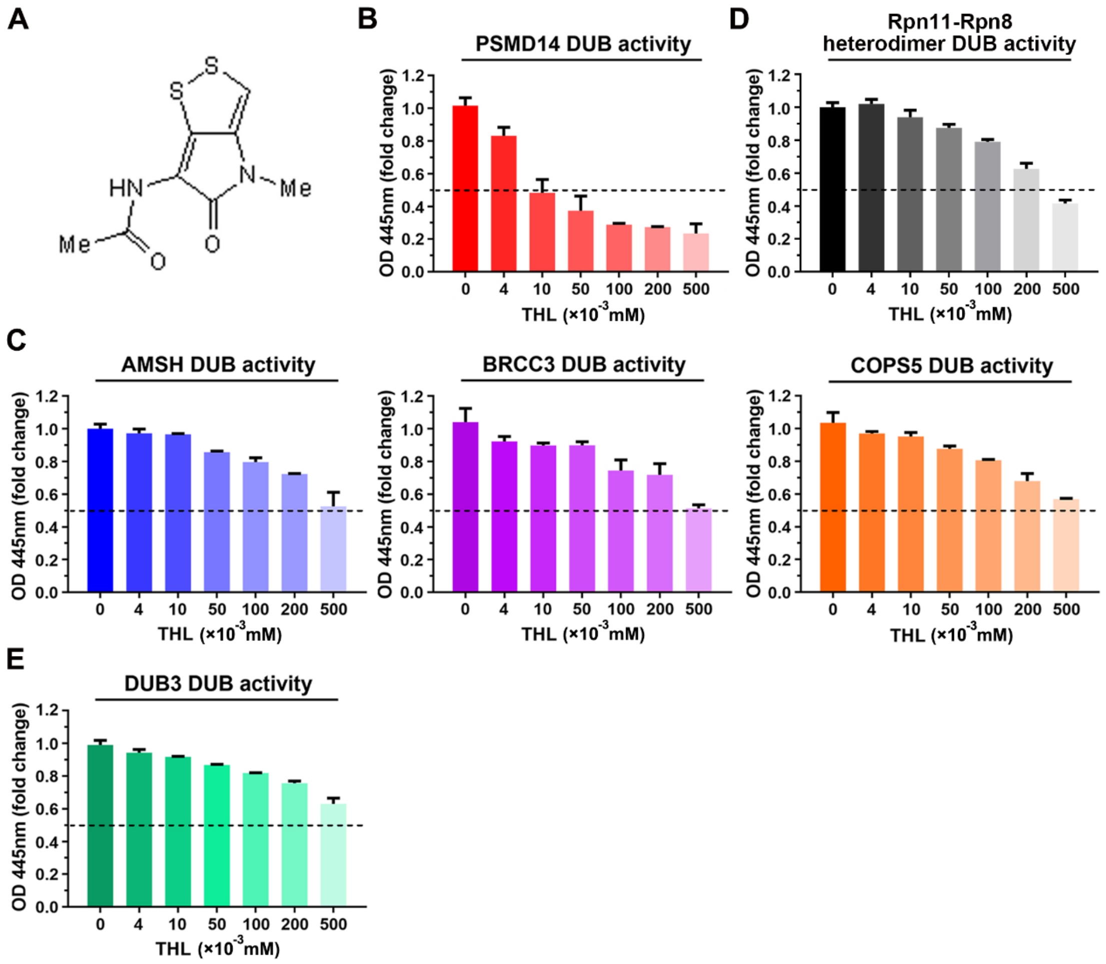

Figure S3. The effect of THL on the activity of DUBs. (A) Chemical structure of Thiolutin. **(B-E)** Recombinant proteins were incubated with THL, followed by the measurement of the absorbance at OD 445 nm to detect DUB activity using Ubiquitin-AMC assay. Data, mean ± SD. THL, Thiolutin.

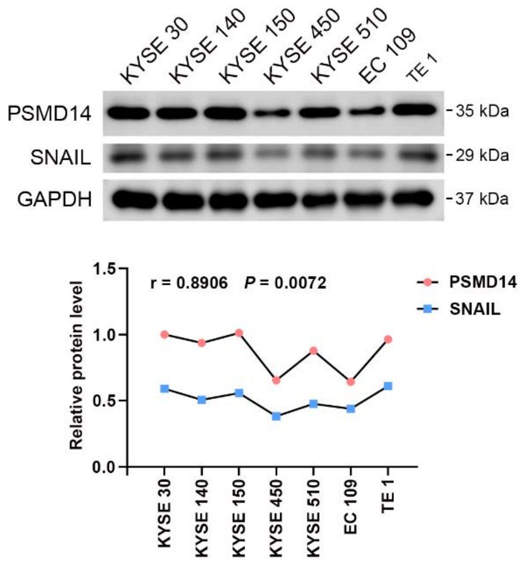

Figure S4. The positive correlation between PSMD14 protein and SNAIL protein among a panel of ESCC cell lines. The protein expressions of PSMD14 and SNAIL were determined by immunoblotting.

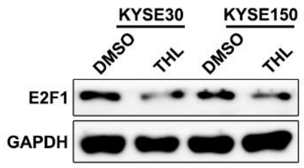

Figure S5. Thiolutin inhibits the expression of E2F1 in ESCC cells. THL, Thiolutin.

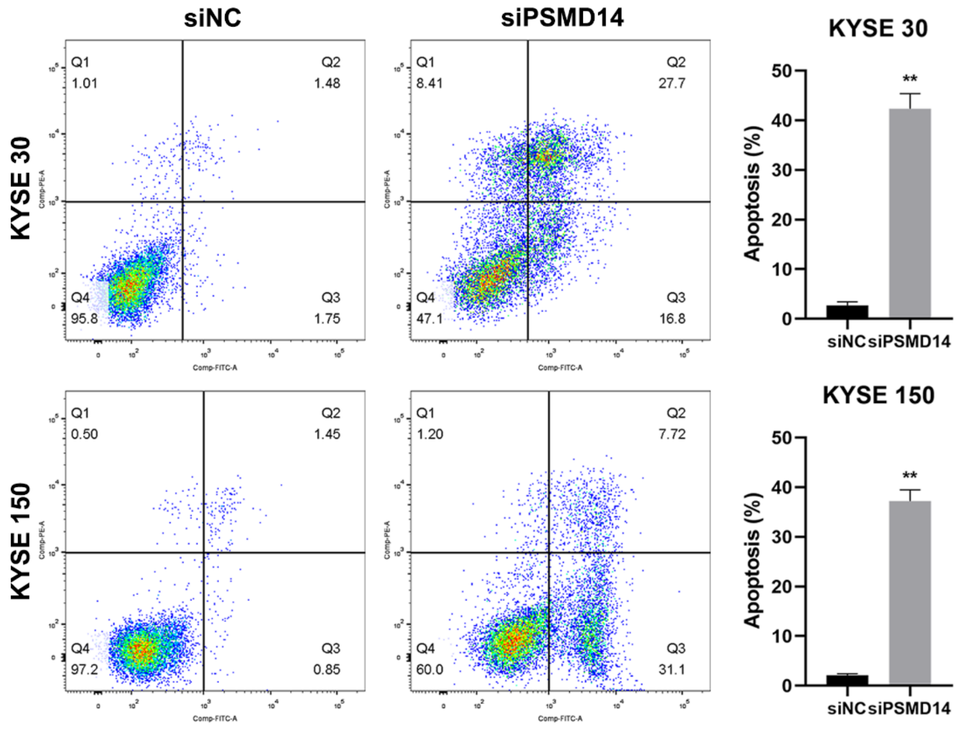

Figure S6. PSMD14 depletion promotes apoptosis of ESCC cells. The apoptosis rates of

KYSE 30 and KYSE 150 cells transfected with siPSMD14 were measured by flow cytometry. Data, mean ± SD, $^{**}P < 0.01$.

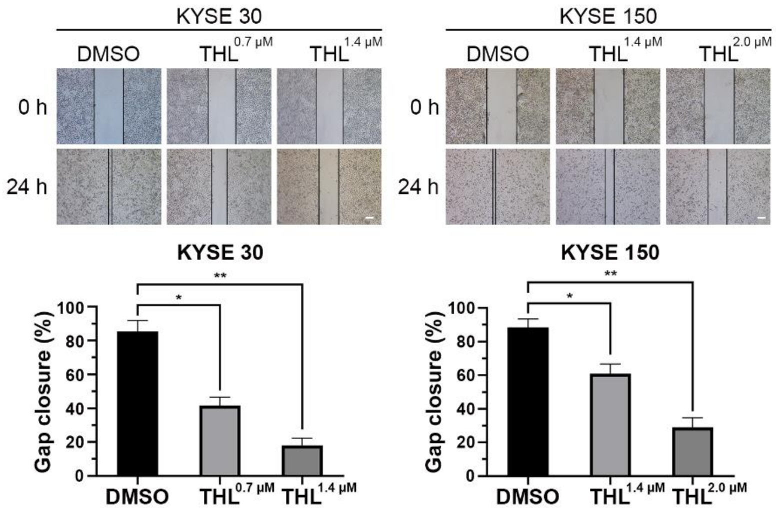

Figure S7. THL impedes the wound-healing process in KYSE 30 and KYSE 150 cells. Representative images of gap at the beginning (0 h) and the endpoint (24 h) of wound healing assay were shown. Scale bar, 200 μm. Data, mean ± SD, $^{*}P < 0.05$, $^{**}P < 0.01$.

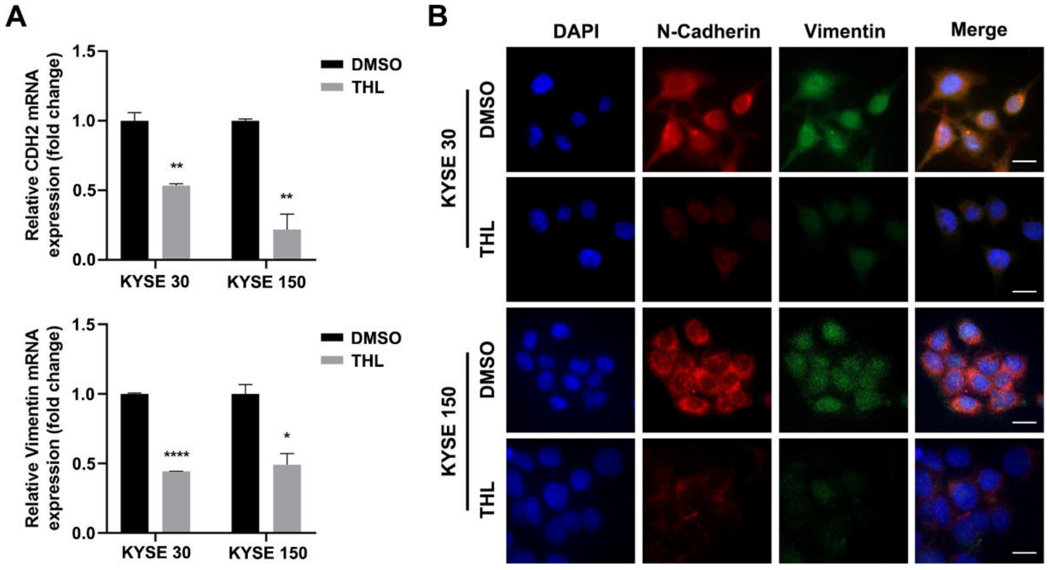

Figure S8. THL reduces the levels of N-Cadherin and Vimentin in ESCC cells. (A) The qPCR results showed that THL decreased the mRNA expression of N-Cadherin (CDH2) and Vimentin in both KYSE 30 and KYSE 150 cells. Data, mean ± SD, $^{*}P < 0.05$, $^{**}P < 0.01$, $^{****}P < 0.0001$. **(B)** The levels of N-Cadherin and Vimentin were assessed by immunofluorescence in ESCC cells treated with THL or DMSO for 24 h. Scale bar, 20 μm.

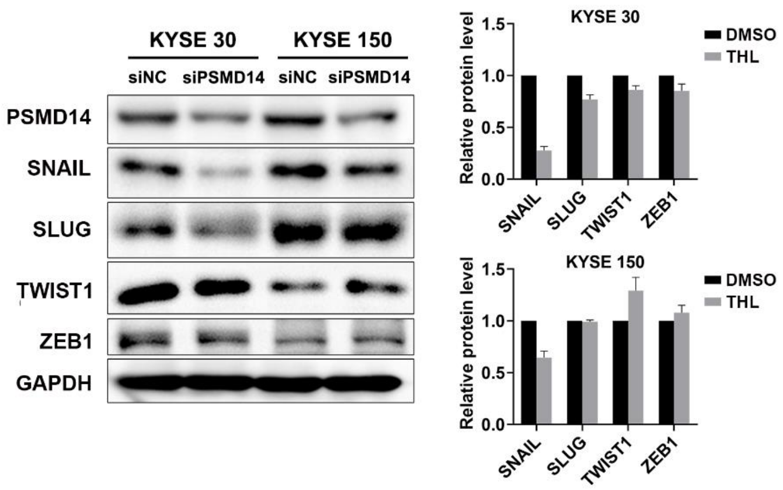

Figure S9. The effect of THL on EMT transcription factors in ESCC cells. The expressions of PSMD14, SNAIL, SLUG, TWIST1 and ZEB1 were detected by immunoblotting assay in indicated treated ESCC cells.

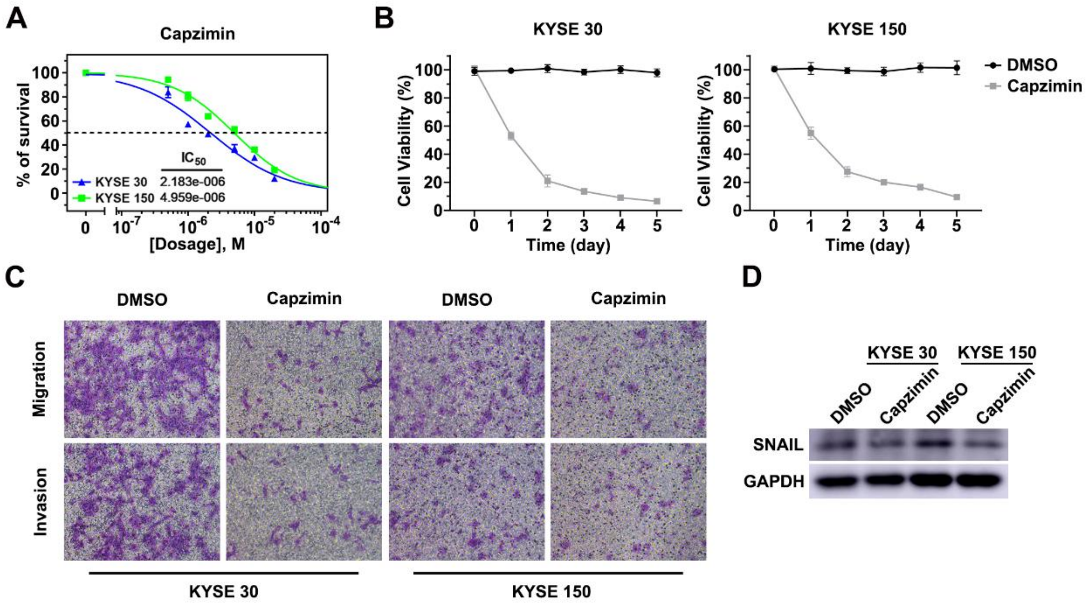

Figure S10. Capzimin inhibits tumor progression of ESCC. (A) The IC_{50} of Capzimin in KYSE 30 and KYSE 150 cell line, respectively. **(B)** The viability of ESCC cells treated with Capzimin for indicated times was measured by MTT assay. Data in **(A)** and **(B)**, mean ± SD. **(C)** Transwell assay results showed that Capzimin (5×10^{-3} mM) significantly hampered migration and invasion of KYSE 30 and KYSE 150 cells. Magnification, 100×. **(D)** KYSE 30 and KYSE 150 cells were exposed to Capzimin (5×10^{-3} mM) for 24 h, then the expression of SNAIL was detected by immunoblotting assay.

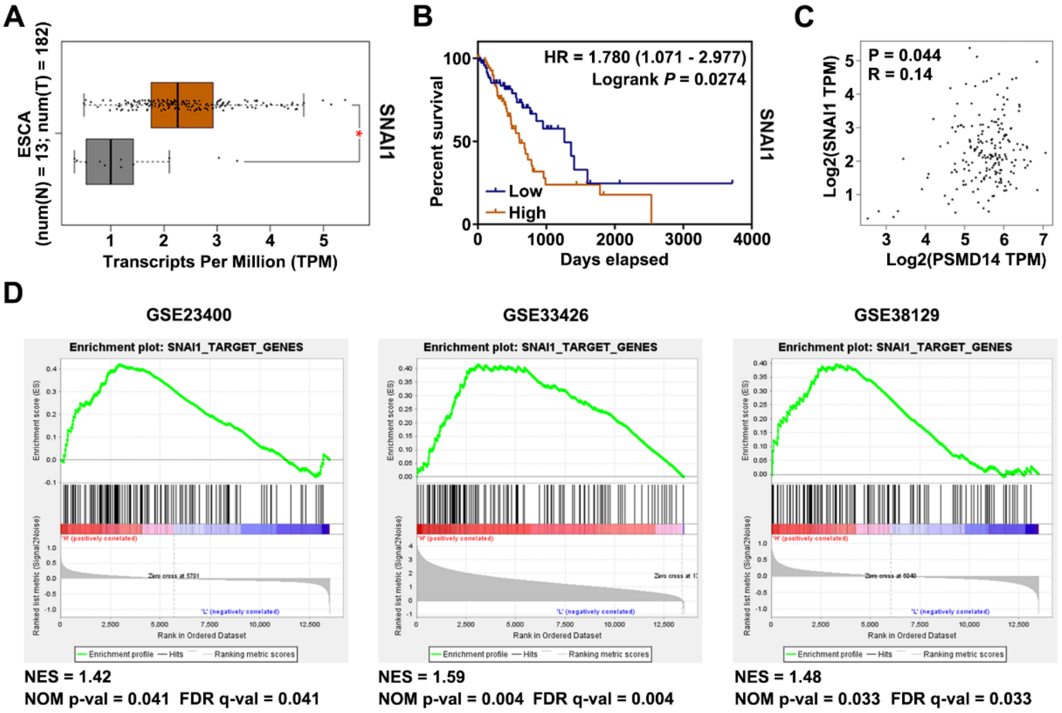

Figure S11. Increased SNAIL predicts poor overall survival in esophageal cancer. (A) TCGA data confirmed that SNAIL increased in esophageal cancer compared with normal tissues. ESCA, esophageal cancer. N, normal tissues. T, tumor samples. Data, mean ± SD, **P* < 0.05. **(B)** Kaplan-Meier plot determined that enhanced SNAIL suggested unfavorite ESCC outcome (HR = 1.780, *P* = 0.0274). **(C)** PSMD14 correlated positively with SNAIL, which was analyzed by GEPIA. **(D)** The enrichment of the signature of SNAIL target genes pathway in the group with high abundancy of PSMD14 from three ESCC cohorts (GSE23400, GSE33426 and GSE38129) was assessed through Gene set enrichment analysis (GSEA). NES, normalized enrichment score. NOM p-val, nominal p-value. FDR q-val, false discovery rates q-value.

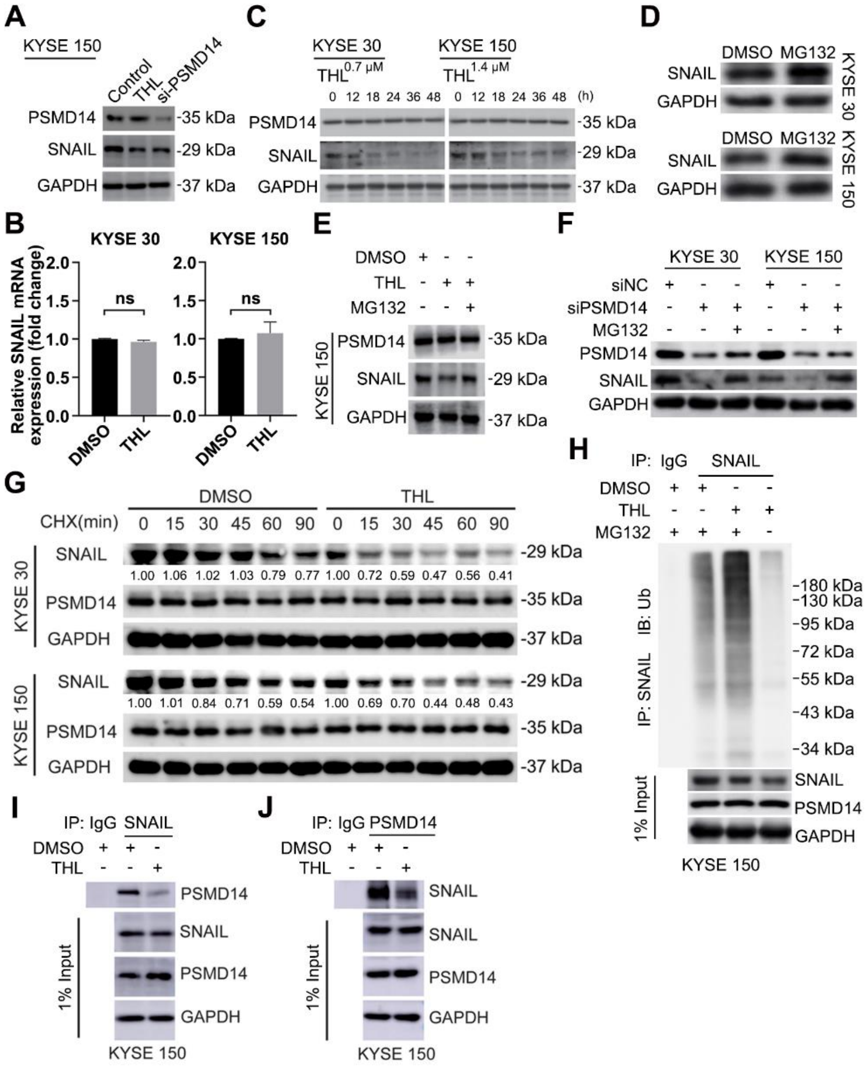

Figure S12. THL induces the ubiquitination and instability of SNAIL. (A) The results of immunoblotting showed that both THL and si-PSMD14 inhibited the protein level of SNAIL in KYSE 150 cells. **(B)** The mRNA level of SNAIL was analyzed by qPCR assay after the treatment of THL for 24 h. Data, mean ± SD, ns, no significance. **(C)** Immunoblotting results indicated that THL significantly suppressed SNAIL protein expression in a time-dependent manner. **(D)** The results of immunoblotting showed that MG132 increased the protein level of SNAIL in ESCC cells. **(E)** The protein levels of PSMD14 and SNAIL were examined by immunoblotting in KYSE 150 cells with indicated treatments. **(F)** Immunoblotting results suggested that MG132 nearly abrogated the inhibition of siPSMD14 on SNAIL protein. **(G)** KYSE 30 and KYSE 150 cells, pretreated with THL or DMSO for 24 h, were exposed to 0.05 mg/ml CHX at the indicated time point for 0, 15, 30, 45, 60, 90 min. The SNAIL protein expression was analyzed by immunoblotting. **(H)** Pretreated with THL or DMSO for 24 h, KYSE 150 cells were incubated with MG132 for another 12 h. The endogenous SNAIL was immunoprecipitated using anti-SNAIL antibody and its poly-ubiquitination level was measured by anti-ubiquitin antibody. 1% input of cell lysates was used to analyze protein levels of PSMD14 and SNAIL. **(I)** KYSE 150 cells were treated with THL or DMSO for 24 h. Cell lysates were immunoprecipitated to pull down PSMD14 by anti-SNAIL antibody. **(J)** Anti-

PSMD14 antibody was used to pull down SNAIL by a co-immunoprecipitation assay, as described in **(I)**.
